# 

**Published:** 1888-03

**Authors:** 


WHOLE NUMBER CCCXX.

MARCH, 1888.

NUMBER 8. VOLUME XXVII.

### THE BUFFALO Medical AND Surgical Journal

ESTABLISHED IN YEAR 1844.

LIBRARY NDEXE S.G.O.

**EDITORS:**

**THOS. LOTHROP, M. D. A. R. DAVIDSON, M. D.**

ASSOCIATE EDITORS:

FLOYD S. CREGO, M. D. CARLTON C. FREDERICK, M. D.
FRED. R. CAMPBELL, M. D. FRANK H. POTTER, M. D.

EDWARD B. ANGELL, M. D., Rochester, N. Y.

